# Retraction: The synthesis and biological activity of marine alkaloid derivatives and analogues

**DOI:** 10.1039/d1ra90083h

**Published:** 2021-02-22

**Authors:** Shiyang Zhou, Gangliang Huang

**Affiliations:** Chongqing Key Laboratory of Green Synthesis and Application, Active Carbohydrate Research Institute, College of Chemistry, Chongqing Normal University Chongqing 401331 China huangdoctor226@163.com; Key Laboratory of Tropical Medicinal Resource Chemistry of Ministry of Education, College of Chemistry and Chemical Engineering, Hainan Normal University Haikou Hainan 571158 China

## Abstract

Retraction of ‘The synthesis and biological activity of marine alkaloid derivatives and analogues’ by Shiyang Zhou *et al.*, *RSC Adv.*, 2020, **10**, 31909–31935, DOI: 10.1039/D0RA05856D.

The Royal Society of Chemistry, with the agreement of the authors, hereby wholly retracts this *RSC Advances* article. Following peer review, during production, the authors were asked to supply an updated manuscript file to correct the referencing format. At this stage, the authors provided a manuscript with substantial content changes. Unfortunately, this was not identified until after publication. As these changes were added after the peer review process, we are retracting this article. The Royal Society of Chemistry apologises that this error was not identified before publication.

Signed: Shiyang Zhou and Gangliang Huang

Date: 11th February 2021

Retraction endorsed by Laura Fisher, Executive Editor, *RSC Advances*

## Supplementary Material

